# Supplementary material for: The Clinical Findings, Pathogenic Variants, and Gene Therapy Qualifications Found in a Leber Congenital Amaurosis Phenotypic Spectrum Patient Cohort
Source: Int J Mol Sci. 2024 Jan 19;25(2):1253. doi: 10.3390/ijms25021253 (PMC10816538; doi:10.3390/ijms25021253)
Supplement: Supplementary file 1 [file ijms-25-01253-s001.zip › ijms-2800734-supplementary.pdf]

Patient Database #	Gene	Syndromic presentation if known	Age at LCA symptom onset or diagnosis if known	Age at which genetic testing was performed	Zygosity	Transcript	Variant(s)			Variant classification per reporting laboratory (ACMG variant classification criteria applied)
23	RPGRIP1		2 years	24 years	Homozygous	NM 020366.3	c.2367+1G>A p.(?)			Likely pathogenic
84	RPE65		2 months	5 months	Homozygous	NM 000329.2	c.271C>T p.(Arg91Trp)			Pathogenic
92	RPE65		3 months	4 years	Homozygous	NM 000329.2	c.271C>T p.(Arg91Trp)			Pathogenic
105	RDH12		7 years	60 years	Compound Heterozygous	NM 152443.2	c.210dup p.(Arg71Glnfs*12)	c.295C>A p.(Leu99Ile)		Pathogenic; pathogenic
107	RDH12		Unknown	40 years	Presumed Compound Heterozygous	NM 152443.2	c.671C>T p.(Thr224Ile)	c.6268C>T p.(Arg1090Cys)		Pathogenic; variant of uncertain significance
179	RPE65		6 months	59 years	Homozygous	NM 000329.2	c.95-2A>T p.(?)			Pathogenic
185	TULP1		Since birth*	23 years	Homozygous	NM 003322.4	c.901C>T p.(Gln301*)			Pathogenic
186	GUCY2D		4 months	6 months	Homozygous	NM 000180.3	c.2983C>T p.(R995Trp)			Likley pathogenic
187	CEP290		2 months	1 year	Presumed Compound Heterozygous	NM 025114.3	c.4723A>T p.(Lys1575*)	c.2251C>T p.(Arg751*)		Pathogenic; pathogenic
192	GUCY2D		Since childhood*	33 years	Homozygous	NM 000180.3	c.65G>A p.(Trp22*)			Pathogenic
233	CRB1		11 months	11 months	Compound Heterozygous	NM 201253.2	c.4039del p.(Thr1347Leufs*5)	c.2570T>G p.(Leu857Arg)		Pathogenic; likely pathogenic
235	CEP290		5 months	7 months	Presumed Compound Heterozygous	NM 025114.3	c.164 167del p.(Thr55Serfs*3)	c.2991+1655A>G p.(?)		Pathogenic; pathogenic
236	CRX		2 years	5 years	Heterozygous	NM 000554.4	c.447dup p.(Ser150Leufs*24)			Pathogenic
238	AIPL1		5 months	9 months	Homozygous	NM 014336	c.834G>A p.(Trp278*)			Pathogenic
243	CEP290**	Joubert syndrome	11 months	7 years	Presumed Compound Heterozygous	NM 025114.4	c.127del4 p.(Glu43Lysfs*6)	c.555del p.(Lys185Asnfs*3)		
244	CRB1		Since birth*	11 years	Homozygous	NM 201253.3	c.1949G>A p.(Trp650*)			Pathogenic
246	NMNAT1		6 months	10 years	Presumed Compound Heterozygous	NM 022787.3	c.6107>G p.(Trp204Gly)	c.769G>A p.(Glu257Lys)		Pathogenic; likely pathogenic
248	CRB1		3 months	5 months	Homozygous	NM 201253.2	c.1949G>A p.(Trp650*)			Pathogenic
249	GUCY2D		7 months	11 months	Compound Heterozygous	NM 000180.3	c.1978C>T p.(Arg660*)	c.2302C>T p.(Arg768Trp)	c.1721G>A p.(Arg574His)	Pathogenic; pathogenic; pathogenic (parental testing confirmed that the p.Arg660* and p.Arg768Trp are in trans)
250	CEP290		Since birth*	4 years	Heterozygous	NM 025114.4	c.2991+1655A>G p.(?)	c.4723A>T p.(Lys1575*)		Pathogenic; pathogenic
251	CEP290		3 months	10 months	Presumed Compound Heterozygous	NM 025114.3	c.2991+1655A>G p.(Cys998*)	c.2771dup p.(Met924Ilefs*4)		Pathogenic; pathogenic
275	CEP290		1 year	24 years	Compound Heterozygous	NM 025114.3	c.2213del p.(Leu738*)	c.2991+1655A>G p.(?)		Pathogenic; pathogenic
276	RPGRIP1		18 years	47 years	Homozygous	NM 020366.3	c.2302C>T p.(Arg768*)			Pathogenic

*Exact age unknown per chart review
**Variants per clinical documentation only
